# Long noncoding RNA PVT1 modulates hepatocellular carcinoma cell proliferation and apoptosis by recruiting EZH2

**DOI:** 10.1186/s12935-018-0582-3

**Published:** 2018-07-11

**Authors:** Jianping Guo, Chong Hao, Congcong Wang, Luo Li

**Affiliations:** 1 Department of Oncology, Maternal and Child Health Care Hospital of Zibo, Zibo, 255029 Shandong China; 2 Scientific Research Office, Zibo Central Hospital, No. 54 West Gongqingtuan Road, Zhangdian District, Zibo, 255000 Shandong China

**Keywords:** Hepatocellular carcinoma, PVT1, EZH2, MDM2, P53

## Abstract

**Background:**

We aimed to figure out the molecular network of PVT1 and EZH2 on hepatocellular carcinoma (HCC) cells growth. We also explored the interaction between PVT1, EZH2, MDM2 and P53.

**Methods:**

Microarray analysis was performed to screen for abnormally expressed genes in HCC tissues and PVT1 was identified as one gene significantly upregulated in HCC. CCK-8 assay, colony formation assay, and flow cytometry detected cell vitality, proliferation and apoptosis, respectively. RIP and RNA pull-down assays were employed to examine the connection between PVT1 and EZH2. The effect of PVT1 on the stability of EZH2 protein and the impact of EZH2 on MDM2 were detected by ELISA. Co-immunoprecipitation assay was used to evaluate the relationship between MDM2 and EZH2. Western blot detected the expression of EZH2, MDM2 and P53.

**Results:**

Up-regulated PVT1 was detected in HCC. Knockdown of PVT1 inhibited HCC cell propagation and promoted apoptotic cells. PVT1 could improve EZH2 protein stability by binding to EZH2 protein but have no significant impact on *EZH2* mRNA expression. EZH2 protein stabilized MDM2 protein expression by binding to MDM2 protein. PVT1 enhanced the protein expression of EZH2 and MDM2 as well as inhibited P53 protein expression.

**Conclusions:**

PVT1 promoted HCC cell propagation and inhibited apoptotic cells by recruiting EZH2, stabilizing MDM2 protein expression and restraining P53 expression.

**Electronic supplementary material:**

The online version of this article (10.1186/s12935-018-0582-3) contains supplementary material, which is available to authorized users.

## Background

As an aggressive malignancies, hepatocellular carcinoma (HCC) results in high mortality of patients [1]. Despite great advances in uncovering molecular mechanisms underlying HCC over the past decades, HCC still menaces patients’ life with high rate of tumor recurrence and metastasis [2]. Therapy such as surgery, radiotherapy and chemotherapy still has limited efficacy because most patients are diagnosed at advantaged stages [3]. Thus, it’s urgent to characterize the pathogenic mechanisms of HCC in order to identify novel targets for HCC.

Long non-coding RNAs (lncRNAs) with more than 200 nucleotides are short of protein coding potential [4]. LncRNAs have been demonstrated to play a critical biological role in carcinogenesis by regulating gene expression [5]. Some publications reported that besides cancer diagnosis and prognosis, lncRNAs can serve as potential target of tumors treatment [6]. In HCC, some differentially expressed lncRNAs have been characterized as tumor promoter or suppressor. For example, linc00052 was down-regulated in HCC, and the upregulation of linc00052 inhibited HCC cells migration and invasion [7]. In contrast, lncRNA PCAT-1 expression was aberrantly up-regulated in HCC, which induced HCC cell invasion and migration [8]. These studies revealed the diversity of lncRNAs impacts on HCC progression.

The plasmacytoma variant translocation 1 gene (PVT1) is a copy number amplification-associated lncRNA [9]. PVT1 functions as an oncogene, which contributes to the phenotype of multiple cancers [10]. For example, PVT1 promoted the growth of non-small cell lung cancer [11]. PVT1 was up-regulated in thyroid tissues and cells, and silenced PVT1 significantly restrained cell propagation and arrested cell cycle at G0/G1 phase [12]. PVT1 has been discovered to be associated with HCC progression in many literatures. Wang et al. elucidated that PVT1 promoted proliferation and cell cycling in HCC cells [13]. Zhang et al. found that PVT1 had high diagnostic value in HCC [14]. Therefore, the functions of PVT1 in HCC need to be further investigated.

The enhancer of zeste homolog 2 (*EZH2*) is a subunit of the multi-enzyme complex polycomb repressive complex 2 and is involved in chromatin compaction and gene repression [15]. *EZH2* was verified to contribute to the aggressiveness of various human cancers [15]. For example, *EZH2* promotes cell proliferation in laryngeal carcinoma [16], inducing cell metastasis in oral cancer [17], increasing cell invasion in endometrial cancer [18] Dysregulation of *EZH2* in HCC has been found in some studies. For instance, up-regulated *EZH2* was measured in HCC tissues, which was positively correlated with tumor grade and clinical stage [19]. Cheng et al. found that downregulation of *EZH2* inhibited HCC cell growth through inhibition of β-catenin signaling [20]. Some studies reported the mechanism of PVT1 and *EZH2*. For example, PVT1 was verified to induce the increase of *EZH2* in gastric cancer [10], thyroid cancer [12] and glioma [21]. However, the connection of PVT1 and *EZH2* in HCC remains unclear.

In conclusion, our study demonstrated the effects of PVT1 and *EZH2* in HCC. In current study, we measured PVT1 expression in HCC, and examined the effects of PVT1 on HCC cell activities. In addition, we performed experiments to confirm the connection between PVT1 and EZH2 as well as MDM2 in HCC cells. Our study showed that PVT1 was highly expressed in HCC tissues and these results highlighted the crucial role of PVT1 in HCC, which may function as a therapies target for HCC.

## Methods

### Tissue specimens

A total of 121 HCC tissue samples and matched non-tumor normal tissue samples were obtained from patients who underwent surgical resection without any form of preoperative chemotherapy and/or radiation therapy at Zibo Central Hospital. All tissues were immediately stored at − 80 °C in liquid nitrogen. Written informed consent was taken from all participants and this study was approved by the ethics committee of Zibo Central Hospital.

### Microarray analysis

The gene microarray hybridization and sample analysis were performed by Ribobio (Guangzhou, China), using the Human 8 × 60 k LncRNA Expression Microarray V3.0 (AS-LNC-M-V3.0, Arraystar Inc., Rockville, USA). Total RNA was quantified with NanoDrop 2000 (Thermo Fisher Scientific Inc., USA). After synthesized with total RNA, cDNA was labeled according to Nimblegen Gene Expression Analysis protocol (Nimblegen Systems, Inc., WI, USA). Data analysis was conducted by NimbleScan (Nim-blegen, USA).

### Cell culture

Three Human HCC cells (HepG2, Huh7, SK-HEP-1, and BEL-7404) and a normal liver cell (HL-7702) were purchased from the BeNa Culture Collection (Beijing, China). HepG2 and SK-HEP-1 in minimum essential medium (MEM, GIBCO BRL, USA) were cultured with 10% fetal bovine serum (FBS, GIBCO BRL, USA); Huh7 was cultured in Dulbecco’s modified Eagle’s medium (DMEM) with 10% FBS (GIBCO BRL); BEL-7404 and HL-7702 were maintained in RPMI1640 medium (GIBCO BRL, USA) supplemented with 10% FBS (GIBCO BRL).

### QRT-PCR

TRIzol Reagent (Invitrogen, Carlsbad, CA, USA) and NanoDrop 2000 (Thermo Fisher Scientific Inc., USA) were utilized to isolate and quantify total RNA. RNA was reversely transcribed to cDNA by using ReverTra Ace qPCR RT Kit (Toyobo, Japan). QRT-PCR was conducted by THUNDERBIRD SYBR qPCR Mix (Toyobo, Japan). Internal controls were U6 and GADPH. 2^−∆∆Ct^ method determined comparative quantification. Primer sequences were exhibited at Table 1.

**Table 1:** Primer sequence

Gene	Sequence
PVT1-F	5′-ATAGATCCTGCCCTGTTTGC-3′
PVT1-R	5′-CATTTCCTGCTGCCGTTTTC-3
GAPDH-F	5′-GGAGCGAGATCCCTCCAAAAT-3′
GAPDH-R	5′-GGCTGTTGTCATACTTCTCATGG-3′
EZH2-F	5′-TTGTTGGCGGAAGCGTG-3′
EZH2-R	5′-TCCCTAGTCCCGCGCAATGTGC-3
pcDNA3.1-PVT1-F	5′-GGGGTACCCTCCGGGCAGAGCGCGTGTG-3′
pcDNA3.1-PVT1-R	5′-CGGGATCCTAGACACGAGGCCGGCCACGC-3′
si-EZH2	5′-AUCAGCUCGUCUGAACCUCUU-3′
si-NC	5′- GGGCCAGACTGGGAAGAAA -3′
si-MDM2	5′-GTGTGTAATAAGGGAGATA-3′
si-PVT1	5′-CAGCCATCATGATGGTACT-3′

### Cell transfection

Si-PVT1, PVT1-pcDNA3.1 and pcDNA3.1 were synthesized by GenePharma (Shanghai, China). Cells were seeded into 6-well plates (1 × 10^6^). Transfection was performed by Lipofectamine 2000 (Life Technologies, USA). Cells transfected with pcDNA3.1 served as NC group; cells transfected with PVT1-pcDNA3.1 was regarded as PVT1 group; cells transfected with si-PVT1 and pcDNA3.1 served as si-PVT1 group.

### CCK-8 assay

8 × 10^3^ HepG2 and Huh 7 cells per well transfected with si-NC or si-PVT1 were incubated into 96 well and cell vitality was assessed by Cell Counting Kit-8 (Biotechwell, Shanghai, China) at 24, 48, 72 and 96 h according to the manual. Absorbance was recorded at 490 nm.

### Colony information assay

Cells transfected with si-NC or si-PVT1 were collected at logarithmic growth phase. Afterwards, cells were placed in a 6-well plate (1 × 10^3^/well) for 2 weeks. After discarding the medium, we used 4% paraformaldehyde to fix cells for 15 min, and added Giemsa solution to stain for 5 min.

### Flow cytometry

Cell apoptosis was assessed by Annexin V-FITC (Keygen, China). Cells were re-suspended in 500 μL binding buffer containing 5 μL propidium iodide (PI) and 5 μL annexin V-FITC. Cells were analyzed by BD Accuri C6 flow cytometer (BD, USA).

### Western blot

Protein samples were harvested from cells to were lysed in RIPA buffer (Beyotime, Shanghai, China). The concentration of protein was determined using Pierce BCA Protein Assay Kit (Pierce, Rockford, IL, USA). After separated by 10% SDS-polyacrylamide gel electrophoresis (SDS-PAGE), proteins were transferred to polyvinylidene difluoride membranes (PVDF, Millipore, USA), blocked with TBST including 5% non-fat skimmed milk. Primary antibodies were added (anti-P53, ab1431, 0.5 µg/mL; anti-GAPDH, ab181603, 1:10,000; anti-*EZH2*, ab191080, 1:500; anti-MDM2, ab38618, 1:1000, Abcam, Cambridge, MA, USA). After washed three times with TBST, second anti-body was added (IgG-HRP, ab7090, 1:2000, Abcam, Cambridge, MA, USA) for 1.5 h. Signal detection was carried out with an ECL system (Life Technology, USA).

### RNA immunoprecipitation (RIP) assay

RIP assay was conducted by the Magna RIP RNA-Binding Protein Immunoprecipitation Kit (Millipore, MA, USA) according to the manual. The supernatants of cell extracts were incubated with treated-beads for 6 h. We used the RIP wash buffer to wash the beads for 6 times. The purified RNA was used for qRT-PCR analysis.

### RNA pull-down assay

PVT1 and the antisense RNA were transcribed by mMESSAGE mMACHINE T7 Kit (Ambion, USA) and RNeasy Mini Kit (Qiagen, Valencia, CA) in vitro, biotin-labeled using Pierce RNA 3′ End Desthiobiotinylation Kit (Thermo Scientific, USA). 1 mg total protein from HepG2 to Huh7 cell extracts were mixed with 50 pmol of biotinylated PVT1 for 1 h, and then added with 60 µL of Streptavidin Beads (Invitrogen) for 1 h. The proteins were resolved by 10% SDS-PAGE, detected by conventional western blot analysis.

### ELISA

After transfected with si-NC, si-PVT1 or si-*EZH2*, HepG2 and Huh7 cells in 24-well plates (2 × 10^5^/well) were added with cyclohexane (CHX, 100 μg/mL, Sigma, USA). Total protein was extracted from cell culture media using RIPA buffer (Beyotime, Shanghai, China), followed by blocking with 5% non-fat PBS for 1 h. After participation of primary antibody for 30 min, cells were added with second antibody for another 30 min and then added with TMB for 15 min. 2 mol/L H_2_SO_4_ was added for 5 min and the optical density was read at 450 nm using a VICTOR3 Microplate Reader (Perkin Elmer; Waltham, MA).

### Co-immunoprecipitation (Co-IP) assay

For co-immunoprecipitation, HepG2 and Huh7 cells at 48-hour post-transfection were washed twice with PBS and lysed in CytoBuster Protein Extraction Reagent (Millipore). Cell lysates were purified with Protein G Plus-Agarose beads (Santa Cruz) and incubated with prepared antibody-conjugated beads overnight. The beads were dissolved in 30 μL SDS-PAGE loading buffer for protein analysis with the indicated antibodies.

### Statistical analysis

GraphPad Prism 6.0 software (GraphPad Software Inc., USA) analyzed all statistics. The measured parameters presented as mean ± SD. Statistical tests for data analysis were two-tailed *t* test and Chi square tests. A probability level of 0.05 was chosen for statistical significance.

## Results

### PVT1 increased in HCC tissues and correlated with clinicopathologic characters of HCC

**Fig. 1:**
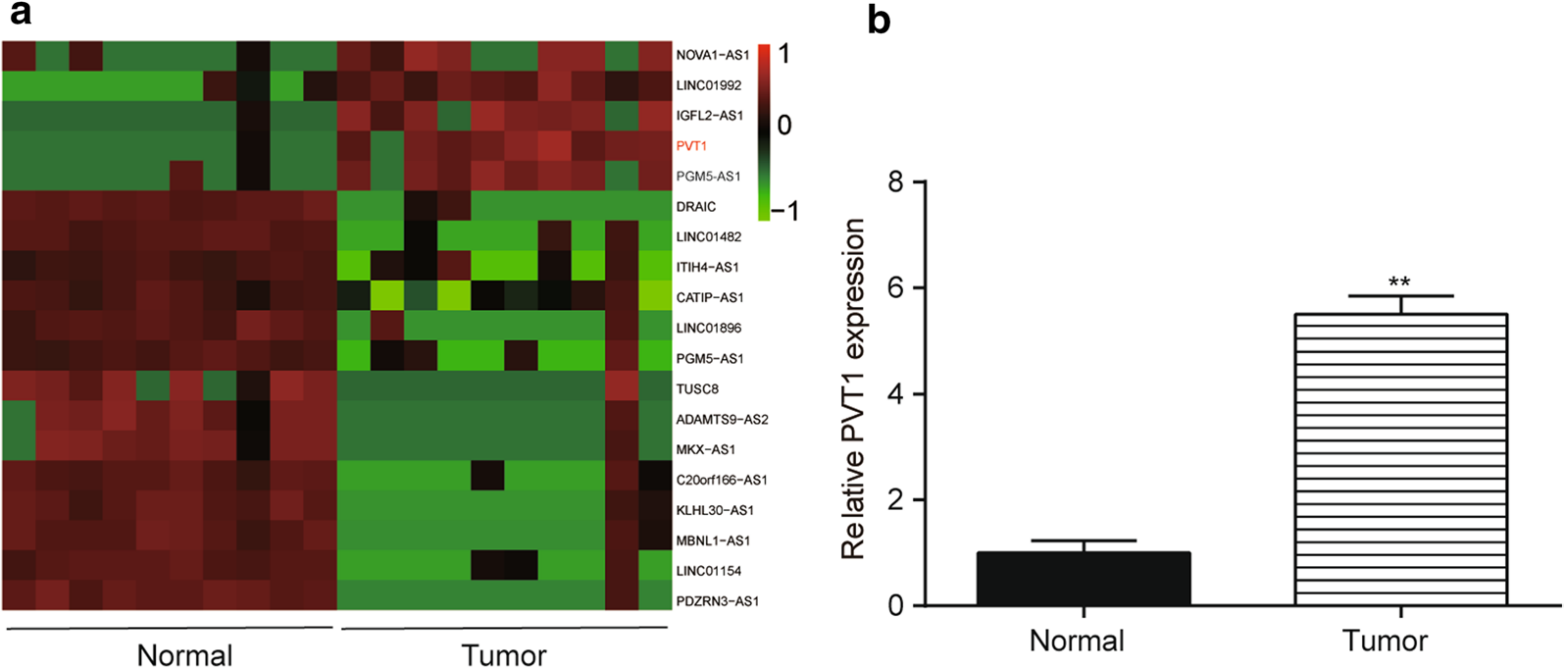
PVT1 was up-regulated in HCC tissues. **a** Heatmap indicated that PVT1 was up-regulated in tumors and down-regulated in normal tissues. **b** QRT-PCR revealed that PVT1 expression in tumors was much higher than that in normal tissues. **P < 0.01, compared with normal tissues

Microarray analysis elucidated that PVT1 was up-regulated in HCC tissues (Fig. 1a, fold change > 2, *P *< 0.05). The results of qRT-PCR confirmed overexpression of PVT1 in HCC tissues (Fig. 1b). Furthermore, through comparing the clinical information of 121 patients, we found that PVT1 expression was linked to tumor size, number, grade and stage (Table 2).

**Table 2:** Clinicopathological correlation of lncRNA PVT1 expression in human HCCs

Variables	No. of cases	PVT1 expression^a^		*P* value*
		Low	High	
Age (years)				0.99
≥ 60	47	28	19	
< 60	74	44	30	
Gender				0.801
Female	65	38	27	
Male	56	34	22	
AFP level (ng/mL)				0.082
≥ 400	60	31	29	
< 400	61	41	20	
Tumor size (cm)				*0.003*
≥ 5	62	29	33	
< 5	59	43	16	
Tumor number				*0.003*
Single	54	40	14	
Multiple	67	32	35	
Tumor grade				*0.001*
G1	59	46	13	
G2 + G3	62	26	36	
Tumor stage				*0.010*
I + II	67	52	15	
III	54	20	34	

*PVT1* plasmacytoma variant translocation 1, *AFP* alphafetoprotein

* For analysis of correlation between PVT1 and clinical features, Pearson’s Chi square tests were used. Results were considered statistically significant at *P *< 0.05 with italics font

^a^ The average expression level was used as the cutoff. Low expression of PVT1 in 72 patients was classified as values below the fold change of 5.44 while high PVT1 expression in 49 patients was classified as values above the level

### Knockdown of PVT1 inhibited HCC cell propagation and promoted apoptotic cells

**Fig. 2:**
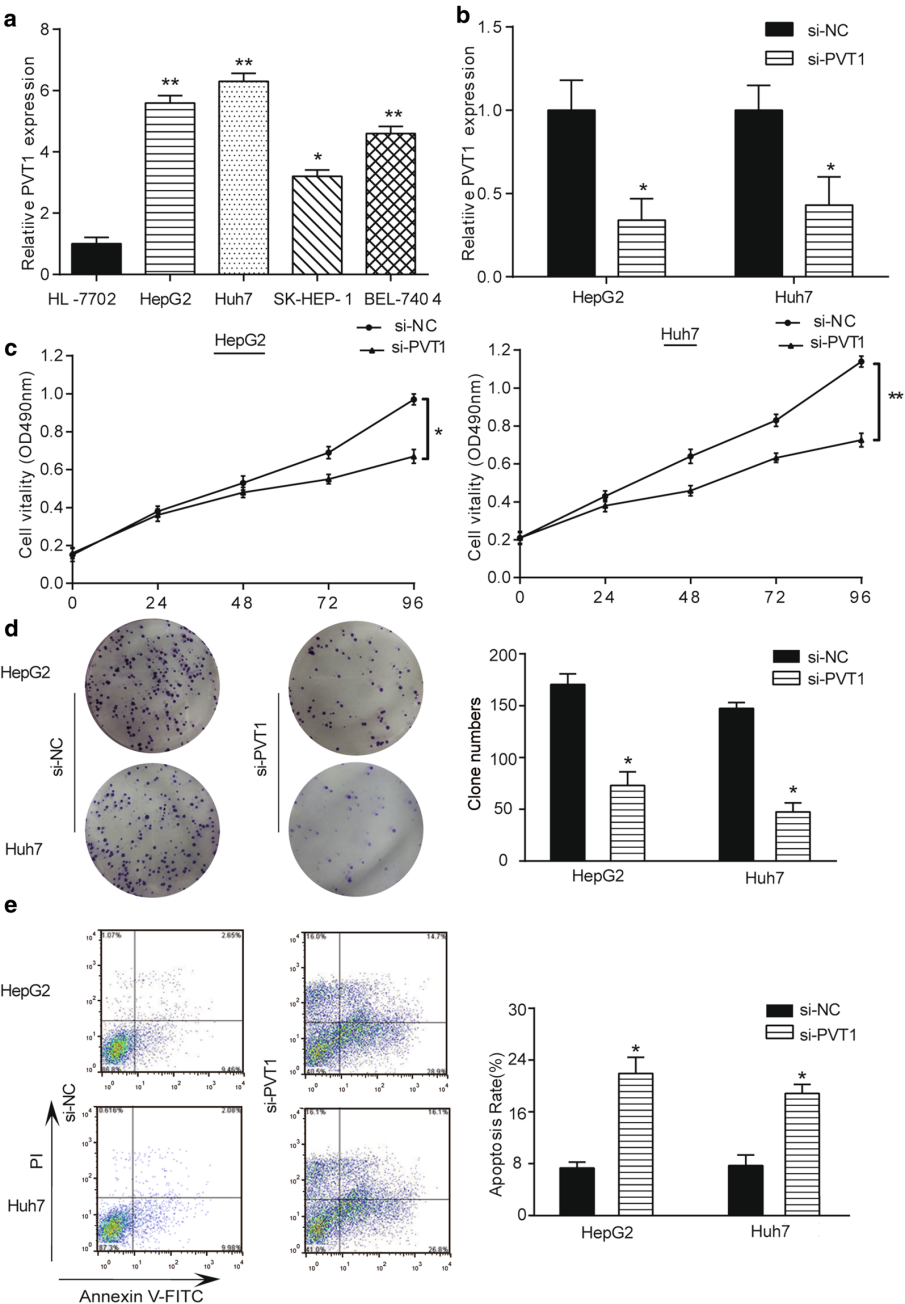
Knockdown of PVT1 inhibited HCC cell proliferation and promoted cell apoptosis. **a** PVT1 was highly expressed in four HCC cell lines (HepG2, Huh7, SK-HEP-1 and BEL-7404) compared with normal cell line (HL-7702). **P < 0.01, *P < 0.05, compared with HL-7702 cell line. **b** PVT1 expression in HepG2 and Huh7 cells transfected with si-PVT1 was lower than that in cells transfected with si-NC. *P < 0.05, compared with si-NC group. **c** Cell vitality of HepG2 and Huh7 cells transfected with si-PVT1 was lower than that in cell s transfected with si-NC detected by CCK-8 assay. *P < 0.01, **P < 0.05, compared with si-NC group. **d** Colony formation assay indicated that the number of colony cells in si-PVT1 group was lower than that in si-NC group. *P < 0.05, compared with si-NC group. **e** Flow cytometry showed that apoptosis rate in si-PVT1 group was higher than that in si-NC group. *P < 0.05, compared with si-NC group

QRT-PCR evaluated PVT1 expression in different HCC cell lines. As shown in Fig. 2a, four cell lines (HepG2, Huh7, SK-HEP-1 and BEL-7404) expressed higher levels of PVT1 compared with normal cell line (HL-7702). PVT1 in HepG2 and Huh7 cell lines exhibited the highest level among four cell line and were selected for further experiments. After knockdown of PVT1 in HepG2 and Huh7 cells, PVT1 expression decreased compared with control group (Fig. 2b, *P *< 0.05). CCK-8 and colony formation assays elucidated that cell vitality and proliferation in si-PVT1 group were attenuated compared with control group (Fig. 2c, d, all *P *< 0.05). Furthermore, knockdown of PVT1 induced apoptosis in HepG2 and Huh7 cells through flow cytometry analysis (Fig. 2e, *P *< 0.05).

### PVT1 improved EZH2 protein stability

**Fig. 3:**
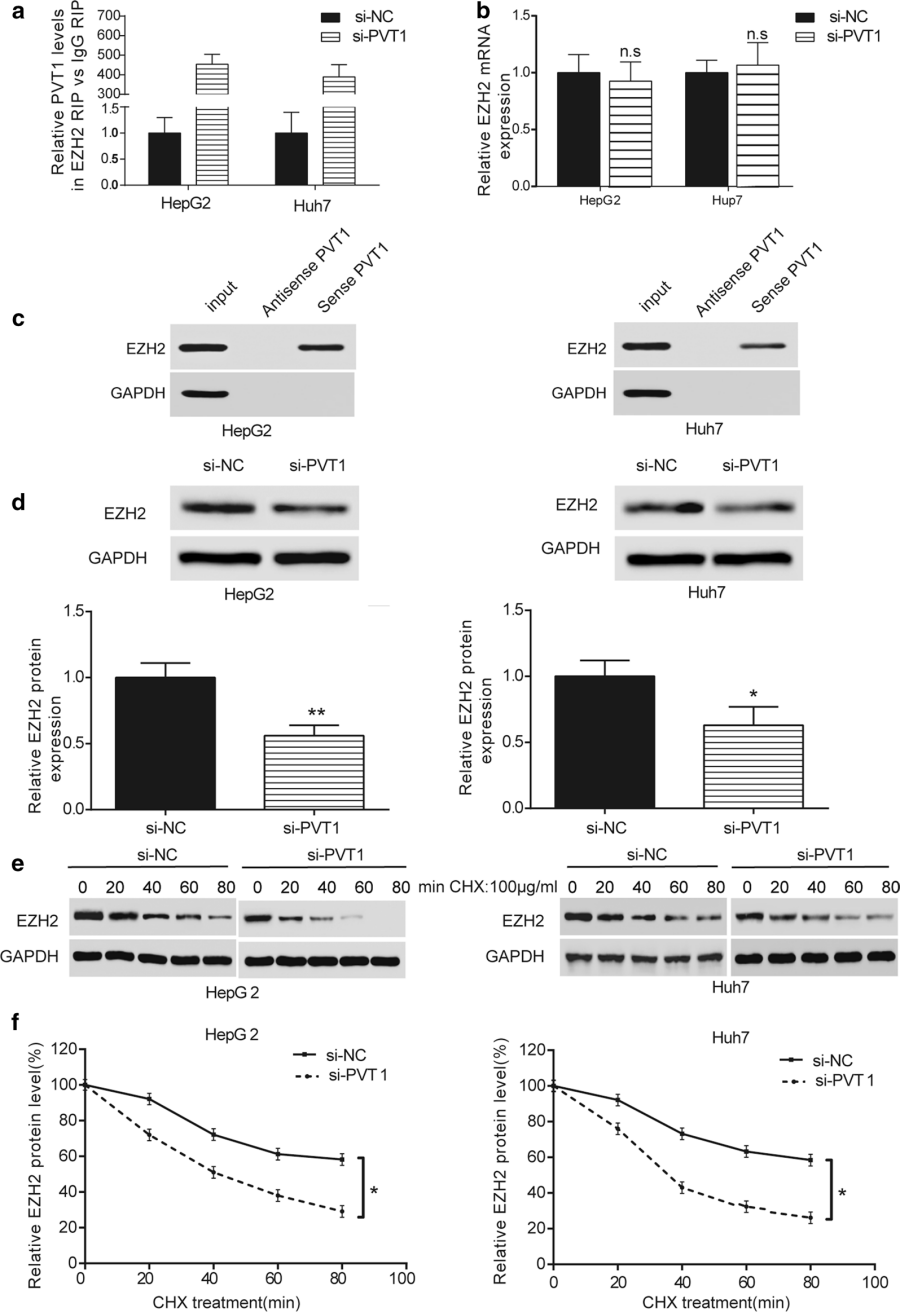
PVT1 could bind to EZH2. **a** RIP assay indicated that PVT1 levels of EZH2 RIP vs IgG RIP in si-PVT1 group was higher than that in si-NC group. **P < 0.01, compared with si-NC group. **b** QRT-PCR indicated that *EZH2* mRNA expression in si-PVT1 group had no significant difference between that in si-NC group. **c** RNA pull-down assay indicated that PVT1 bound to EZH2. **d** Western blot revealed that EZH2 protein expression in si-PVT1 group was lower than that si-NC group. *P < 0.05, compared with si-NC group. **e** Western blot indicated that after added with 100 μg/mL CHX, si-PVT1 had greater inhibitory effect on EZH2 protein expression with the increase of time. **f** ELISA assay revealed that EZH2 protein expression in si-PVT1 group was lower than that in si-NC group with the increase of time after added with 100 μg/mL CHX. *P < 0.05, compared with si-NC group

RIP experiments were conducted in HepG2 and Huh7 cells, which revealed PVT1 enrichment in *EZH2*-RNA participation compared with si-NC group (Fig. 3a, *P *< 0.05). In addition, knockdown of PVT1 had no impact on *EZH2* mRNA expression (Fig. 3b) and RNA pull-down assays in HepG2 and Huh7 cells revealed that EZH2 interacted with PVT1 (Fig. 3c, *P *< 0.05). However, PVT1 was not directly bound to MDM2 or P53 (Additional file 1: Figure S1). In contrast, si-PVT1 inhibited EZH2 protein expression (Fig. 3d, *P *< 0.05). Western blot and ELISA assays demonstrated that si-PVT1 decreased the EZH2 protein stability (Fig. 3e, f, *P *< 0.05). Taken together, PVT1 could improve EZH2 protein stability but have no significant impact on *EZH2* mRNA expression.

### EZH2 protein stabilized MDM2 protein expression

**Fig. 4:**
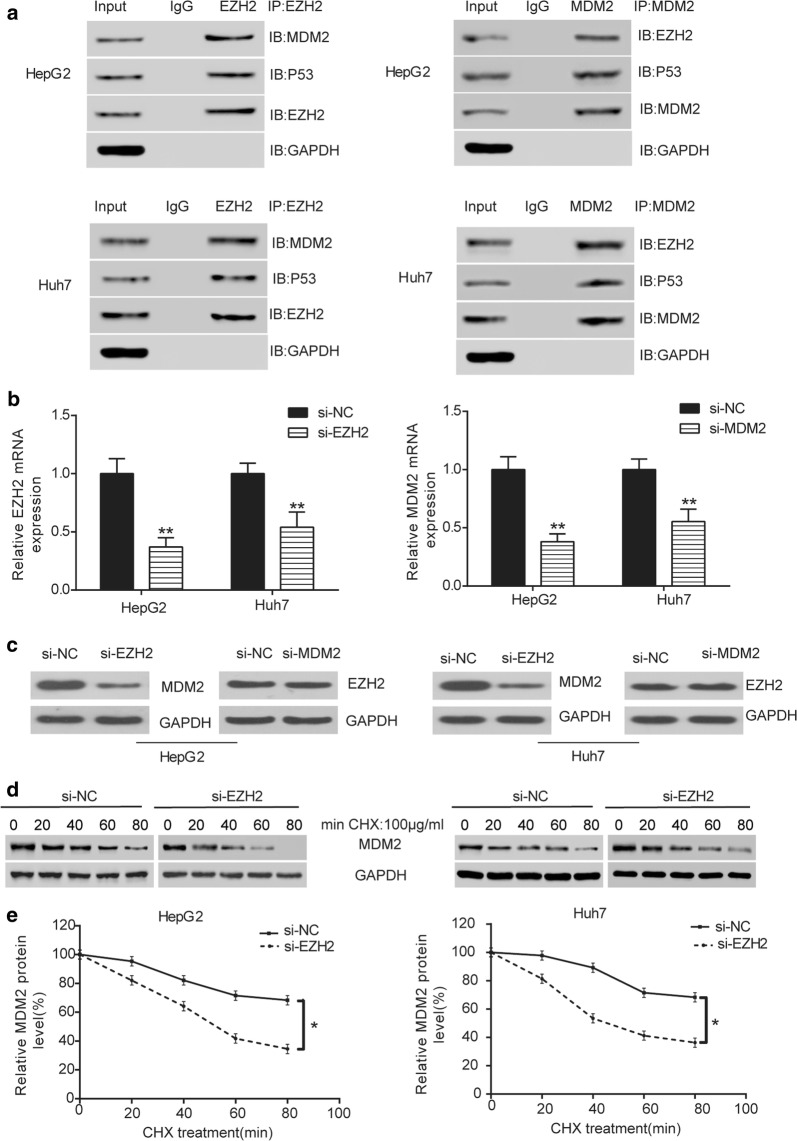
EZH2 protein stabilized MDM2 protein expression. **a** Co-IP assay indicated that EZH2 bound to MDM2. **b** QRT-PCR indicated that *EZH2* mRNA in si-*EZH2* group was lower than that in si-NC group. **P < 0.01, compared with si-NC group. **c** Western blot showed that MDM2 protein expression in si-*EZH2* group was lower than that in si-NC group, while EZH2 protein expression in si-MDM2 group had no significant difference with that in si-NC group. **d** Western blot indicated that after added with 100 μg/mL CHX, si-*EZH2* had greater inhibitory effect on MDM2 protein expression with the increase of time. **f** ELISA assay revealed that MDM2 protein expression in si-*EZH2* group was lower than that in si-NC group with the increase of time after added with 100 μg/mL CHX. *P < 0.05, compared with si-NC group

In Co-IP assay, we found that EZH2 could bind to MDM2 (Fig. 4a). To further study the interaction between EZH2 and MDM2, we successfully transfected HepG2 and Huh7 cells with si-*EZH2* and si-MDM2 (Fig. 4b, *P *< 0.05). Measured by western blot, knockdown of EZH2 decreased MDM2 protein expression, whereas knockdown of MDM2 had no significant impact on EZH2 protein expression (Fig. 4c). Meanwhile, the MDM2 protein half-life decreased after knockdown of EZH2 (Fig. 4d, e, *P *< 0.05). Therefore, EZH2 protein improved the stability of MDM2 protein.

### PVT1 enhanced EZH2 and MDM2 expression as well as inhibited P53 protein expression

**Fig. 5:**
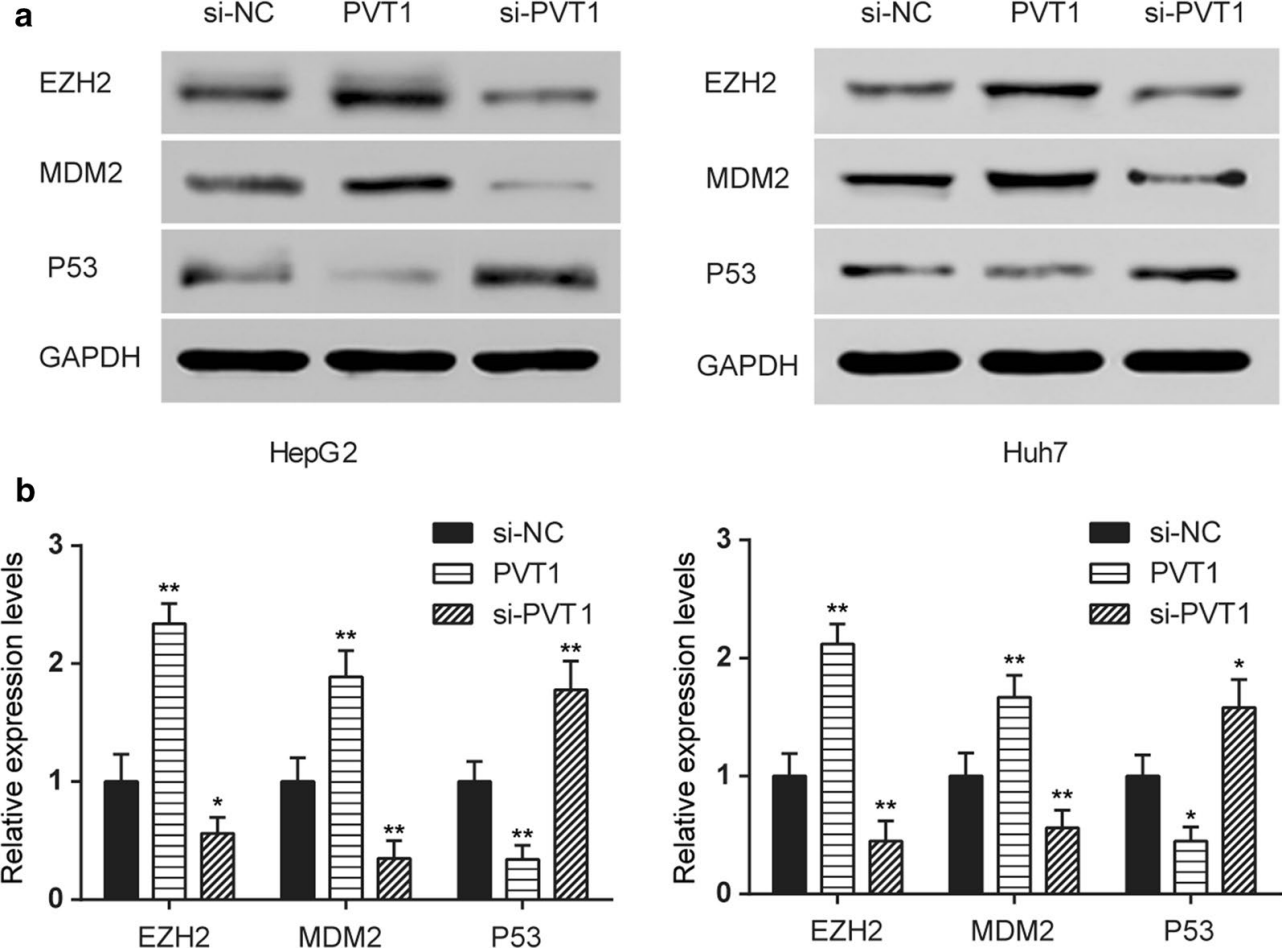
PVT1 enhanced the protein expression of EZH2 and MDM2 as well as inhibited P53 protein expression. **a**, **b** Western blot demonstrated that overexpression of PVT1 improved the protein expression level of EZH2 and MDM2 as well as inhibited P53 protein expression in HepG2 and Huh7 cells. In contrast, knockdown of PVT1 inhibited the protein expression level of EZH2 and MDM2 as well as promoted P53 protein expression

After transfected with pcDNA3.1-PVT1 and si-PVT1 in HepG2 and Huh7 cells, western blot was employed to determine the protein expression of EZH2, MDM2 and P53. The results elucidated that overexpression of PVT1 improved the protein expression of EZH2 and MDM2 as well as inhibited P53 protein expression. In contrast, knockdown of PVT1 had the opposite effects (Fig. 5).

## Discussion

Mounting evidence has highlighted the critical roles of lncRNAs in HCC cellular process [22]. We performed series experiments to investigate the function of PVT1 on HCC. First, we confirmed PVT1 overexpression in HCC, and inhibition of PVT1 suppressed HCC cell propagation and promoted apoptotic cells. Second, we employed RIP, RNA pull-down and ELISA assays to examine the connection of PVT1 and EZH2, finding that PVT1 could improve EZH2 protein stability by binding to EZH2. Meanwhile, the results of co-IP assay revealed that EZH2 protein stabilized MDM2 protein expression. Additionally, PVT1 enhanced the protein expression of EZH2 and MDM2 as well as inhibited P53 protein expression in HCC cells.

LncRNAs are involved in regulating biological functions and gene expression in physiological and pathological contexts, such as cancer [23]. Growing evidence has clarified the functions of different lncRNAs in HCC, including lncRNA-PVT1 [24]. PVT1 functioned as an oncogenic lncRNA in multiple types of cancers [25]. PVT1 overexpression has been identified as predictor for many carcinomas [9]. For example, PVT1 knockdown significantly inhibited prostate cancer growth in vivo and in vitro and promoted cell apoptosis [26]. Zhuang et al. found that PVT1 silencing inhibited bladder cancer cell growth and induced apoptosis [27]. Based on previous studies, we speculated that PVT1 might have impacts on HCC cell process. In current study, PVT1 in HCC was aberrantly higher. After knockdown of PVT1, inhibited HCC cell proliferation was inhibited while cell apoptosis was enhanced. Consistently, Gou et al. also found that PVT1 was increased in HCC, which promoted cell proliferation and invasion in HCC [6]. Lan et al. demonstrated that PVT1 promoted proliferation, invasion and migration in HCC cells by regulating miR-186-5p [28].

*EZH2* has been thought to contribute to malignant transformation due to its role in regulating fundamental cellular processes [29]. Previously, some studies have reported the correlation between PVT1 and EZH2. For example, PVT1 induced lung adenocarcinoma progression through LATS2/MDM2/P53 pathway suppressed by EZH2 [30]. Kong et al. revealed that PVT1 recruited EZH2 and contributed to gastric cancer growth [10]. In our study, we performed RIP, RNA pull-down and ELISA assays to examine the relationship between PVT1 and EZH2. All experiments demonstrated that PVT1 improved EZH2 protein stability by binding to EZH2. Furthermore, we found that EZH2 protein stabilized MDM2 protein expression by binding to MDM2 detected by co-IP assay. Previous study has claimed that as a key negative regulator of the tumor suppressor p53, MDM2 is recruited to target gene promoters by EZH2 [31]. We also employed western blot to measure the effects of PVT1 on EZH2, MDM2 and P53. The results indicated that PVT1 enhanced the protein expressions of EZH2 and MDM2 as well as inhibited P53 protein expression. In this study, we first clearly elucidated the molecular mechanism underlying PVT1, EZH2, MDM2 and P53 in HCC.

However, some concerns still existed in our study. In current study, we only investigated the effects of PVT1 on P53 expression, while the molecular network between EZH2, MDM2 and P53 need to be further explored. On the other hand, the pathway of PVT1, EZH2 and P53 in HCC should be taken into consideration.

## Conclusions

In summary, our findings suggested that PVT1 had positive effects on HCC cell growth. Additionally, PVT1 stabilized the protein expression of EZH2 and MADM2. PVT1 might be a potential therapies target of HCC treatment. Our experiments may improve the diagnostic ability of biomarkers for HCC in clinical practice.

## Additional file

**Additional file 1: Figure S1.** PVT1 could not directly bind to MDM2 or P53. (A) RNA pull-down assay indicated that PVT1 could not directly bind to MDM2 or P53.

## Abbreviations

Co-IP: co-immunoprecipitation
CHX: cyclohexane
*EZH2*: enhancer of zeste homolog 2
HCC: hepatocellular carcinoma
lncRNAs: long non-coding RNAs
PI: propidium iodide
PVT1: plasmacytoma variant translocation 1 gene
RIP: RNA immunoprecipitation
SDS-PAGE: SDS-polyacrylamide gel electrophoresis
