# Supplementary figures and images for: Long noncoding RNA PVT1 modulates hepatocellular carcinoma cell proliferation and apoptosis by recruiting EZH2
Source: Cancer Cell Int. 2018 Jul 11;18:98. doi: 10.1186/s12935-018-0582-3 (PMC6042336; doi:10.1186/s12935-018-0582-3)

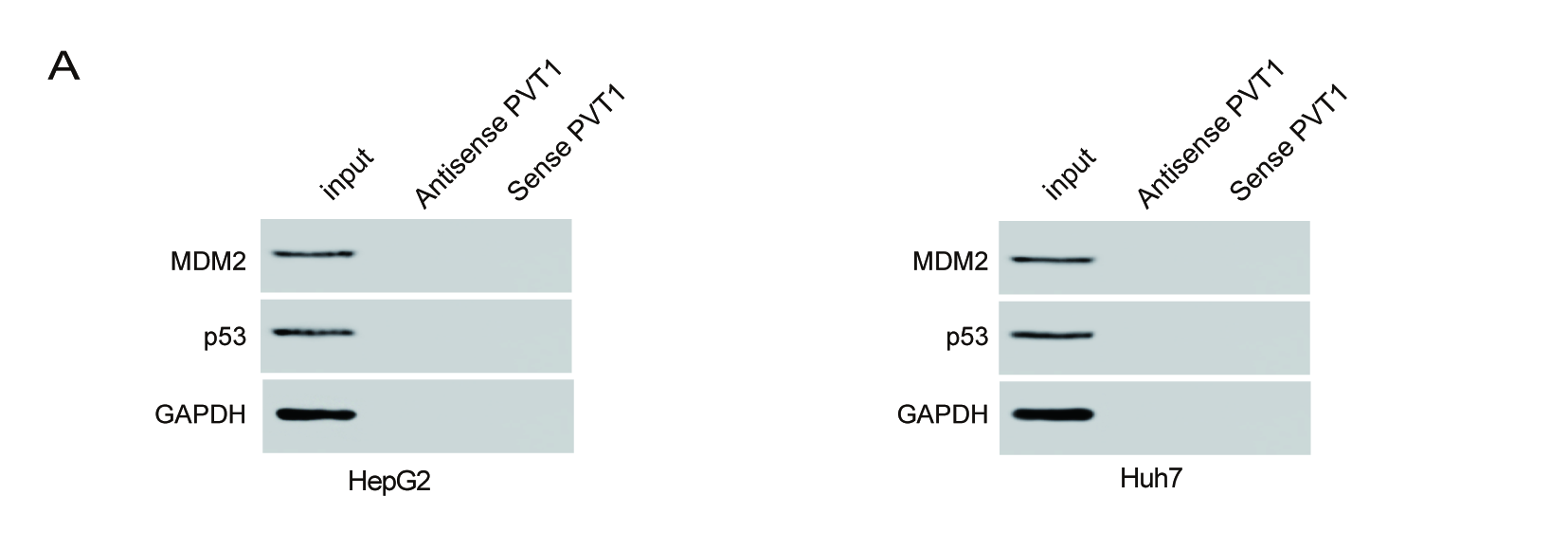

Supplement: Supplementary file 1 — Additional file 1: Figure S1. PVT1 could not directly bind to MDM2 or P53. (A) RNA pull-down assay indicated that PVT1 could not directly bind to MDM2 or P53. [file 12935_2018_582_MOESM1_ESM.tif]
